# Virtual Reality for Supporting the Treatment of Depression and Anxiety: Scoping Review

**DOI:** 10.2196/29681

**Published:** 2021-09-23

**Authors:** Nilufar Baghaei, Vibhav Chitale, Andrej Hlasnik, Lehan Stemmet, Hai-Ning Liang, Richard Porter

**Affiliations:** 1 Department of Natural and Computational Sciences Massey University Auckland New Zealand; 2 Otago Polytechnic Auckland International Campus Auckland New Zealand; 3 Auckland Institute of Studies Auckland New Zealand; 4 Department of Computing Xi’an Jiaotong-Liverpool University Suzhou China; 5 Department of Psychological Medicine University of Otago Christchurch New Zealand

**Keywords:** virtual reality, mental health, depression, anxiety, CBT

## Abstract

**Background:**

Mental health conditions pose a major challenge to health care providers and society at large. The World Health Organization predicts that by 2030, mental health conditions will be the leading cause of disease burden worldwide. The current need for mental health care is overwhelming. In New Zealand, 1 in 6 adults has been diagnosed with common mental disorders, such as depression and anxiety disorders, according to a national survey. Cognitive behavioral therapy (CBT) has been shown to effectively help patients overcome a wide variety of mental health conditions. Virtual reality exposure therapy (VRET) might be one of the most exciting technologies emerging in the clinical setting for the treatment of anxiety and depression.

**Objective:**

This study aims to investigate the virtual reality (VR) technologies currently being used to help support the treatment of depression and anxiety. We also aim to investigate whether and how CBT is included as part of VRET and look at the VR technologies and interventions that have been used in recent studies on depression and anxiety.

**Methods:**

We performed a scoping review. To identify significant studies, we decided to use already aggregated sources from the Google Scholar database. Overall, the goal of our search strategy was to limit the number of initial results related to VR in mental health to only a relevant minimum.

**Results:**

Using our defined keywords, Google Scholar identified >17,300 articles. After applying all the inclusion and exclusion criteria, we identified a total of 369 articles for further processing. After manual evaluation, 34 articles were shortlisted; of the 34 articles, 9 (26%) reported the use of CBT with VR. All of the articles were published between 2017 and 2021. Out of the 9 studies, CBT was conducted within a VR environment in 5 (56%) studies, whereas in the remaining 4 (44%) studies, CBT was used as an addition to VRET. All 9 studies reported the use of CBT either in vivo or in a virtual environment to be effective in supporting the treatment of anxiety or depression.

**Conclusions:**

Most studies demonstrated the use of VR to be effective for supporting the treatment of anxiety or depression in a range of settings and recommended its potential as a tool for use in a clinical environment. Even though standalone headsets are much easier to work with and more suitable for home use, the shift from tethered VR headsets to standalone headsets in the mental health environment was not observed. All studies that looked at the use of CBT either in vivo or in a virtual environment found it to be effective in supporting the treatment of anxiety or depression.

## Introduction

### Background

Mental health conditions are a major challenge for society, health care providers, and health systems, with the recent COVID-19 pandemic only worsening these pre-existing conditions [1]. Mental health services are struggling to meet the needs of users and fail to reach large proportions of those in need of care. The World Health Organization (WHO) predicts that by 2030, mental disorders will be the leading cause of disease burden worldwide [2]. The WHO has also estimated that anxiety disorders have cost the global economy approximately US $1 trillion per year in lost productivity costs [3]. Between 75% and 85% of people with mental disorders remain untreated in low-income countries, with almost 1 million people taking their lives each year. In addition, according to the WHO, 1 in 13 people is affected by anxiety worldwide, with specific phobias, major depressive disorders, and social phobias being the most common. Barriers to effective care include a lack of resources, lack of trained health care providers, and social stigma associated with mental disorders [2].

According to a recent New Zealand health survey, 1 in 6 New Zealand adults has been diagnosed with common mental disorders such as depression and anxiety disorders. Māori and Pacific adults (indigenous population) have higher rates of being diagnosed with depression and anxiety than the rest of the population. There is also a certain level of societal stigma attached to mental health problems, preventing some people from accessing the available resources [4].

Mood disorders and anxiety disorders are closely linked, and individuals who develop depression often experience an anxiety disorder earlier in life. Indeed, individuals with depression often experience symptomology similar to that characteristic of anxiety disorders, including nervousness, irritability, disturbed sleep or appetite, and poor concentration, among other symptoms [5].

### Cognitive Behavioral Therapy

Cognitive behavioral therapy (CBT) is among the therapies commonly available for treating anxiety and depression. It is supported by many guidelines as a first-line treatment for mood and anxiety disorders [6,7]. CBT is a type of psychotherapeutic treatment that helps people learn how to identify and change destructive or disturbing thought patterns that have a negative influence on behavior and emotions [8]. It is the most empirically supported therapy and has been shown to effectively help patients overcome a wide variety of mental health conditions, including anxiety and depression [8].

### Exposure Therapy

Exposure therapy, a form of behavioral therapy, has become increasingly popular in recent years with both mental health consumers and treatment professionals as one of the most effective treatments for phobic anxiety disorders. Exposure therapies are usually conducted through in vivo exposure (IVE) or imaginal exposure (IE). In IVE therapy, the patient is deliberately exposed to the feared object or situation in the real world. It is often used in the treatment of phobias or anxieties [9,10]. Despite being an effective treatment, some IVE therapies may require to be conducted in public, thereby risking patient confidentiality, or are too expensive or face difficulty in replicating the feared scenarios (eg, fear of flying). To help overcome these IVE limitations, IE therapy can be an alternative approach to trigger the feared situations through imagination. However, for IE therapy to be effective, the patient must be ready and willing to spend time and effort analyzing their thoughts and feelings.

### Virtual Reality and Virtual Exposure Therapy

The use of virtual reality (VR) in health care was pioneered by Hoffman et al [11,12] in the early 2000s, with a VR gaming system called SnowWorld that was able to reduce pain perception during burn wound care in both adolescent and adult patients. VR is the use of computer modeling and simulation that enables a person to interact with an artificial 3D visual environment or other sensory environments. VR systems typically comprise the following components:

- Graphics rendering units: the computer hardware to compute the virtual scene and render it to a frame buffer, ready to be sent to a display device. This is typically a high-end graphics computer.
- 3D stereo display units: it operates as the interface from the computer to the user. Visual information is often presented via large projection-based displays or head-mounted displays (HMDs).
- Tracking system: serves as the interface between the user and the computer. Modern HMDs include integrated head tracking, thereby allowing the user to move their head and change their visual perspective in the virtual environment accordingly.
- Other interfaces include joysticks or sensory gloves, which provide tactile feedback.
- Examples of recent popular VR devices include Oculus Quest 2, HTC Vive, and Sony PlayStation VR.

VR might be one of the most exciting, emerging technologies that is rapidly gaining traction in the treatment of anxiety and depression [13-15]. VR exposure therapy (VRET) is a modern type of exposure therapy that follows the same procedures as traditional exposure therapy, with the only difference being that the feared objects or situations are rendered within a virtual environment. A virtual environment provides a greater degree of control for the therapists to customize, reproduce, and tweak several treatment parameters according to the patient’s needs. Such a level of customization cannot be achieved in traditional exposure therapy. Risk of privacy intrusion is reduced as everything is confined to a virtual environment. Furthermore, VRET is considered less frightening than IVE therapy according to the patients [16].

VR applications and VRETs have also been shown to be effective in the treatment of a variety of other mental health conditions, such as specific phobias (acrophobia and arachnophobia) or social anxiety disorder (SAD), autism, panic disorder, posttraumatic stress disorder, substance abuse, or addiction disorders (alcohol and gambling) [9,10,17-23]. Thus, this study presents findings from a scoping review on state-of-the-art VR therapies for supporting the treatment of anxiety and depression.

### Objective

A scoping review methodology specified by Arksey and O’Malley [24] was followed in this study. In this scoping review, we aim to investigate two questions: (1) what VR technologies and interventions have recently been studied in depression and anxiety disorders and (2) whether and how CBT is included and used as part of VRET (within the VR environment or in addition to a VR intervention)?

As technology advances rapidly, it is critical to understand the current state-of-the-art technologies being used, especially in interdisciplinary fields such as VR in mental health. Thus, to answer the first question, we will study VR technology, types of interventions, participants’ interactions during the intervention, and how the virtual environments were created.

It is known that CBT is an effective tool used for the treatment of several mental health problems [8]. Common VR interventions, such as VRET, may be sufficient to treat anxiety or depression; however, the combination of these interventions with CBT has been an understudied area. There is currently much less literature that specifically explores the effectiveness of CBT used in combination with common VR interventions. Thus, the second question systematically explores the use of CBT along with VRET and provides a detailed review of how CBT was used, whether it was used inside a virtual environment or in addition to VRET, and the effectiveness of this type of methodology in the treatment of anxiety or depression.

## Methods

### Search Strategy and Eligibility Criteria

We defined the search strategy specifically suited to find the most relevant papers that used VR to support and enhance the outcome of mental health issues, particularly focusing on anxiety and depression.

### Databases Searched

We used Google Scholar as the primary source of materials included in this research. Google Scholar provides a simple and quick way to search across a variety of disciplines, databases, and journals. To limit the number of studies relevant to our research questions (RQs), we defined specific eligibility criteria and search terms. The goal of our search strategy was to limit the number of results related to our RQs to only a relevant minimum.

### Search Terms

The search terms were discussed among the research team and were defined as *Anxiety*, *Depression*, and *Virtual Reality*. Sometimes, *virtual reality* can also be acronymized as *VR*; however, the full term was exclusively used during the search to avoid any potential conflicts with other terms that may use the VR acronym (eg, *voice recognition*). These defined search terms also reflect the most used keywords in the studies relevant to our RQs.

### Eligibility Criteria

As information technology is evolving rapidly in terms of hardware and software related to VR, we decided to limit the year of publication to 2017 to identify state-of-the-art technologies. Therefore, the main article dates were set from 2017 to 2021. Google Scholar advance search allows the selection of articles using only a limited set of criteria. When we used the search filter criteria *anywhere in the article* with the defined search terms *Virtual reality*, *Depression*, and *Anxiety*, the Google Scholar search yielded 17,300 results. On the basis of our search strategy goal, we decided to use a combination of three search requests that were compliant with the following rules:

- All search terms must be present in the article.
- All search terms must be present in the title of the article (not only in the article body).
- The article publication year must be within a specific range from 2017 to 2021.

With these rules in place, we were able to execute three separate search requests using the following combination of search terms:

- *Virtual reality* AND *anxiety*
- *Virtual reality* AND *depression*
- *Virtual reality* AND *depression* AND *anxiety*

Some of the additional exclusion criteria were defined as follows:

- Duplicates, version updates, or written in a language other than English
- Swot analysis, thesis and citations, systematic reviews, or no significant reported results
- Anxiety or depression as a secondary aspect or induced due to an illness

A detailed explanation of the search strategy is provided in Multimedia Appendix 1.

### Study Selection

The search strategy yielded 369 articles overall. The first step included the removal of duplicates, which was found to be 6.5% (24/369) of articles, thus reducing the number of articles to 345. A total of 4 authors screened the titles of the 345 articles for relevance. If a decision could not be made regarding the relevance of an article, then the abstract was taken into consideration. If the authors still lacked certainty, then the full text of the article was reviewed to reach the final decision.

In total, 15.4% (53/345) of articles were excluded because of nonrelevancy as determined by the authors upon screening, making the number of eligible articles 292. The nonrelevant articles included out-of-context studies, such as those not targeting anxiety or depression. Few nonrelevant articles targeted anxiety or depression but relied on technologies other than VR. The last screening step required articles to meet the defined eligibility criteria, which were assessed upon a full-text review. A total of 34 articles were confirmed for the scoping review that met the eligibility criteria.

**Figure 1:**
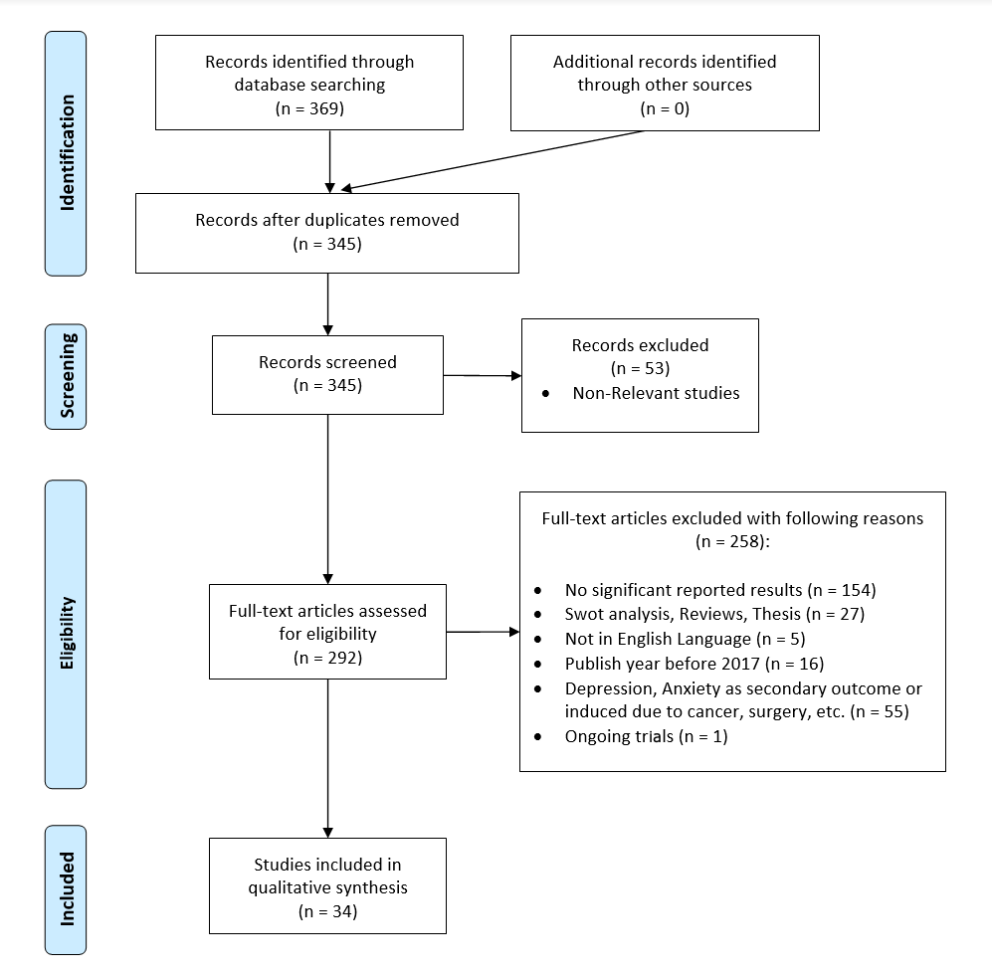
Literature screening and selection flowchart following PRISMA (Preferred Reporting Items for Systematic Reviews and Meta-analyses) guidelines.

This study selection process is depicted in Figure 1.

An in-depth breakdown of the study selection process is explained in Table 1.

**Table 1:** The reasons for exclusions and the number of studies excluded and selected for final review^a^.

Study ID and search terms			Articles, n (%)
**Total collected**			369 (100)
	1	Virtual reality AND anxiety	298 (80.8)
	2	Virtual reality AND depression	62 (16.8)
	3	Virtual reality AND depression AND anxiety	9 (2.4)
**Total excluded**			335 (100)
	1	Excluding nonrelevant studies	53 (15.8)
	2	Excluding duplicities and version updates	24 (7.2)
	3	Excluding documents with no significant reported results	154 (46)
	4	Excluding swot analysis, thesis and citations, and system reviews	27 (8.1)
	5	Excluding documents not in the English language	5 (1.5)
	6	Excluding documents published before 2017	16 (4.8)
	7	Excluding documents with anxiety or depression as a secondary aspect or anxiety or depression induced because of cancer and surgery	55 (16.4)
	8	Excluding documents with ongoing trials	1 (0.3)

^a^Total selected: 100% (34/34).

### Data Extraction

One of the authors performed the data extraction process, whereas the data validity and accuracy were checked by the remaining authors. All articles were downloaded as full text and maintained in a database shared among all authors. The following data were extracted:

1. Publication year and authors
2. Demographics such as sample size, age distribution, and gender ratio
3. Methodology (eg, study design, was CBT included, duration of study, and number of sessions)
4. VR hardware and software details such as type of headset, toolkit used, what was the VR scenario, and how the VR environment was designed
5. Key findings concerning the effectiveness of VR in supporting the treatment of depression and anxiety and the role and significance of CBT, if used

The extracted data allowed us to generate information related to our RQs, especially in determining the experimental setup and significance of CBT. Moreover, the data provided useful insights into the development of the statistics of the articles used in the review.

## Results

### Publications Statistics

A total of 34 articles were selected for this scoping review (Multimedia Appendix 2 [14,15,25-56]). Articles with no available information were discarded during the plotting.

Multimedia Appendix 3 shows most articles published in the year 2019 [25-36], comprising 35% (12/34) of the total articles. Articles published in 2018 [37-43] and 2020 [15,44-49] were of equal proportions (7/34, 21%), whereas those published in 2017 [14,50-53] were the third highest (5/34, 15%). Recent publications from early 2021 [54-56] were also selected (3/34, 9%).

### Demographics

Multimedia Appendix 4 shows the participant’s average age distribution. The age distribution is particularly dominated by the two age groups of 21-30 years and 31-40 years, comprising 67% (21/31) of the total articles. The lowest average age group was 0-10 years, whereas the highest average age group was 71-80 years, indicating that the participants across all age groups were recruited for VR support in tackling depression or anxiety.

Multimedia Appendix 5 depicts the distribution of total participants. Most of the articles (13/34, 38%) included <10 participants [26,31-34,36-38,40-42,45,55], whereas two studies had >90 participants [35,54].

**Figure 2:**
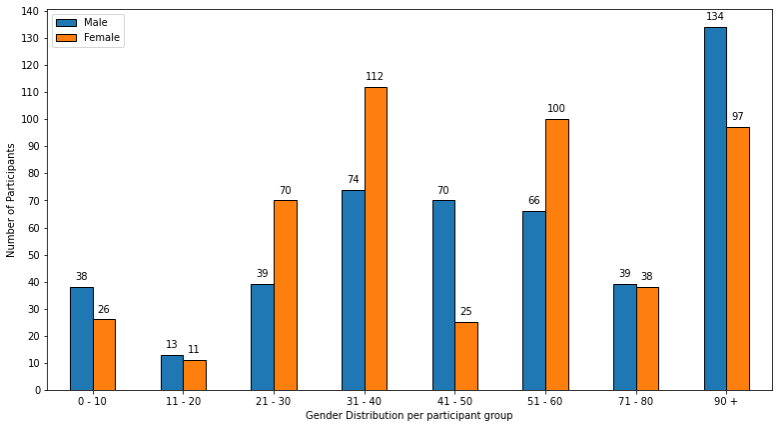
Gender distribution of participants.

Similarly, the gender distribution of the participants is shown in Figure 2. The total number of female and male participants was 479 and 473, respectively, indicating an equal gender ratio among the participants.

### Clinical Conditions

Multimedia Appendix 6 illustrates the count of clinical conditions per publication. Overall, most studies centered around one or the other type of anxiety condition (25/34, 74%) compared with depression (4/34, 12%). SAD was the most studied, with 3% (9/34) of studies focusing on using VR therapy solely for patients with SAD [29,34,37,41,47,50,52-54]. Generalized anxiety disorder (6/34, 18%) [27,28,39,42,46,49] was next, followed by public speaking anxiety (4/34, 12%) [14,31,51,56]. The rest of the studies aimed at specific clinical conditions such as paruresis [40], exam anxiety [48], driving-related [38] or car passenger anxiety [30], disruptive behavior in the classroom [45], or social anxiety in children with autism spectrum disorder [33].

Fewer studies (4/34, 12%) focused on using VR therapy exclusively for the treatment of individuals with depression [15,26,36,43]; 15% (5/34) of studies targeted multiple conditions such as stress [25,35] or posttraumatic stress disorder [44,55] or panic disorder [32], in addition to anxiety and depression.

### Investigating the RQs

#### RQ 1: What VR Technologies and Interventions Are Currently Being Used in Studies on Depression and Anxiety?

To answer this question, we developed a robust data extraction process, which is explained in the earlier section. We reviewed the articles and extracted details such as types of VR interventions, plotted in Multimedia Appendix 7; types of VR headsets, shown in Multimedia Appendix 8; additional monitoring software and hardware, details about what the participants did during the intervention, and number of sessions, depicted in Multimedia Appendix 9; and how long the interventions lasted.

The most frequent type of VR intervention was VRET (standard VR scene), which was used in 26% (9/34) of studies [25,31,34,40,41,47,53,55,56]. Interestingly, 6% (2/34) of studies used behavior therapy in addition to VRET, using commercial simulator software in the treatment of SAD [41] and public speaking anxiety [31]. It was followed by CBT inside VR (5/34, 15%) [26,28,37,50,54] and VR application (5/34, 15%) [30,33,35,43,52] interventions. Approximately 6% (2/34) of studies demonstrated a unique use of VR applications using music therapy (where participants had to sing a song in a virtual hall) [33] and art therapy (where participants performed activities using a tilt-brush VR application) [35]. VR music therapy was used in the treatment of social anxiety in adolescents with autism spectrum disorder. Other interventions applied were VR games or exercises [15,45,46,49], CBT in addition to VR [14,27,38,39], and 360° VR videos [36,44,48,51]. Approximately 9% (3/34) of studies used different types of VR interventions, such as visuo-haptic–based multimodal feedback VR system [32], VR-based neurofeedback therapy [42], and a VR task-tracking avoidance behavior [29].

The most popular VR headset among the articles was HTC Vive (6/34, 18%), whereas Oculus Rift was the next most frequently used VR headset (4/34, 12%). Approximately 9% (3/34) of studies used Gear VR; different types of VR simulators; and Cave Automatic Virtual Environment–like systems, one used Leap Motion [32] and one used Windows Mixed Reality Headsets [36]. A regular feature among most studies was the external monitoring of additional data such as heart rate, eye movement, or electroencephalogram readings during the intervention or pre- and postintervention.

A wide range of VR environments or scenarios was observed. A workplace environment or job interview was the most common virtual environment for participants with SAD. A conference room or classroom was usually used for public speaking anxiety, whereas virtual nature exposure was a common scenario seen in studies focusing on both anxiety and depression. An interesting virtual environment of underwater world exploration was used in the treatment of state anxiety and disruptive classroom behavior, wherein the movement was controlled through participants’ breathing using a VR biofeedback game intervention [45].

Multimedia Appendix 9 shows wide variability in the number of sessions that the participants underwent for VR therapy. A single session was used in most studies (8/34, 24%). Notably, one of the studies had a total of 108 trial sessions, which was an outlier as the range of sessions was observed from 1 to a maximum of 17. The session time ranged from 2-90 minutes, depending on the type of VR intervention.

A recent study by Jeong et al [54] aimed to identify the number of sessions that were sufficient for a successful VR intervention in the treatment of SAD, concluding 9-10 sessions or possibly fewer sessions (5-6) of VR-based CBT can be effective in the treatment of SAD. Increasing the number of sessions above this provided minimal additional benefits.

#### RQ 2: Is CBT Included as Part of VRET and, if so, How Is CBT Used (Within the VR Environment or in Addition to VR Intervention)?

To answer the second question, we systematically reviewed the articles and extracted relevant data that explored the use of CBT along with other VR interventions. We then formulated a review table (Table 2) that described the CBT methodology in terms of how CBT was used, what was the VR experiment, and the key findings.

Approximately 26% (9/34) of studies mentioned the use of CBT with other interventions. Only those studies that explicitly mentioned CBT were selected. Of the 9 studies, 3 (9%) focused on the treatment of SAD and 2 (6%) focused on generalized anxiety disorder. One study targeted both SAD and generalized anxiety disorder. One study mentioned the use of CBT for depression. CBT inside a VR environment was the most used (5/34, 15%); 12% (4/34) of studies used CBT in addition to VRET. Approximately 9% (3/34) of studies made use of commercial software to expose participants to the virtual environment [38,39,50]. Notably, there was no common hardware or software across all studies, except the use of an HMD. Total participants varied from 2 to 115.

All studies reported that the use of CBT was associated with a reduction in symptoms of anxiety or depression, with one study even reporting that conducting CBT inside a virtual environment was more effective than CBT with IVE on the primary outcome measure of anxiety symptoms in a randomized controlled trial (RCT). It was also found to be more practical by therapists than conducting CBT with IVE [50].

**Table 2:** Review of CBT^a^ studies.

ID	Condition	CBT type	VR^b^ toolkit	VR scenario and total participants	Summary of findings
Anderson et al [14]	Public speaking anxiety	CBT and VR	—^c^	Participants gave a speech in front of the increasing number group in a virtual conference room, classroom, and auditorium (n=28).	Participants showed statistically significant improvement on all self-report measures from pretreatment to follow-up.
Stamou et al [26]	Postnatal Depression	CBT inside VR	—	Participants were exposed to a series of virtual stressors, whereas at the same time, they were asked to tidy up the virtual house (n=6).	All participants reported feeling better, more relaxed and with improved mood, better self-esteem, and improved sleep and appetite.
Guitard et al [27]	Generalized anxiety disorder	CBT and VR	A 6-side CAVE^d^-like system and wireless motion tracking	Three standardized VE^e^ of an emergency room (n=11), an apartment (n=15), and a student room (n=2)	The standardized VE induced significant anxiety. No difference was found between standardized VE and imagined scenario.
Geraets et al [28]	Generalized and social anxiety disorder	CBT inside VR	Head-mounted display (Sony HMZ-T1) and joystick	Virtual street, bus, cafe, and supermarket environments were available. Patients tested their beliefs and feedback was given on cognition and behavior (n=15).	Two patients dropped out of treatment. Social anxiety and quality of life improved at posttreatment. At follow-up, depressive symptoms decreased, and social anxiety was maintained.
Kovar I [37]	Social anxiety disorder	CBT inside VR	HTC Vive+ controllers	Public speaking, a telephone call from a random institution, criticism of their appearance, and a job interview or refusal (n=10)	Most significant improvement in the length of fluent speech. VRET^f^ improved reaction speed by 204.8 seconds. No effect in job interview VE.
Zinzow et al [38]	Driving-related anxiety	CBT and VR	Drive Safety CDS^g^-250 driving simulator. Three 19-inch LCD^h^ screen displays	Lane keeping straight, changing, and mirrors, speed control straight, pedals and stopping, functional object detection–basic, turning left and right (n=8)	Hyperarousal in driving situations declined by 69%, aggressive driving declined by 29%, and risky driving declined by 21%.
Tarrant et al [39]	Generalized anxiety disorder	CBT inside VR	SPSS software, Gear VR 19-channel EEG^i^, and Brain Master^j^	A mindfulness meditation by Story Up VR (n=12)	The VR meditation significantly reduced subjectively reported anxiety.
Bouchard et al [50]	Social anxiety disorder	CBT inside VR (RCT^k^)	Virtually Better, eMagin z800 head-mounted display, and InterSense InertiaCube motion tracker	Eight VEs such as a meeting room, job interview, introducing oneself, and facing criticism situations (n=59)	Conducting CBT with in virtuo exposure was effective and more practical for therapists than CBT with in vivo exposure.
Jeong et al [54]	Social anxiety	CBT inside VR	Desktop or mobile version with monitor of eye movement, speaking time, and heart rate	Some VEs were classroom, auditorium, job interview, train, and cafe (n=115)	Short-term VR-based individual CBT of 9-10 sessions may be effective. Minimal benefit if extended.

^a^CBT: cognitive behavioral therapy.

^b^VR: virtual reality.

^c^Not applicable.

^d^CAVE: cave automatic virtual environment.

^e^VE: virtual environment.

^f^VRET: virtual reality exposure therapy.

^g^CDS: Clinical Driving Simulator.

^h^LCD: liquid crystal display.

^i^EEG: electroencephalogram.

^j^BrainMaster Technologies, Inc.

^k^RCT: randomized controlled trial.

## Discussion

### Principal Findings

VR therapy has been used widely for treating a variety of mental health conditions. This scoping review covered 34 articles, which used VR for the treatment of various syndromes of anxiety and depression. It was observed that most studies demonstrated a reduction in symptoms of anxiety or depression with the use of VR. Furthermore, most studies had a follow-up session post intervention to record the effects of the therapy on whether the anxiety or depression improvements were maintained in the participants. Most suggest effectiveness and acceptability in a range of clinical settings.

To gain a deeper understanding of the participants, we performed an exploratory data analysis that visualized the participants’ demographics such as their average age groups (Multimedia Appendix 4), gender ratio (Figure 2), or the total number of participants (Multimedia Appendix 5) involved in the studies. The exploratory data analysis revealed that the two most common average age groups among participants were the young to middle-aged adults of 21-30 years and 31-40 years. The data as depicted in Multimedia Appendix 5 show that there is a lack of research on the older as well as the younger population, with only a handful of studies covering these age groups. Moreover, visualizing the gender distribution in Figure 2 showed that there was almost no gender bias present across the studies as the gender ratio of males (473) to females (479) was almost the same. Interestingly, Multimedia Appendix 5 shows that the most frequent sample size consisted of only 1-10 total participants, suggesting that there is a need to cover a larger sample size for conclusive proof of the presented results.

Although standalone VR headsets are becoming more affordable to obtain, easier to work with, and more suitable for home use, the shift from tethered VR headsets to standalone headsets in mental health studies has not yet been observed. Manufacturers such as Sony, Samsung, Google, HTC, and Microsoft have heavily invested in their own VR products; however, based on our study, HTC Vive and Oculus Rift were the most frequently used headsets. In terms of software assets, studies generally used Unity 3D, and none of the studies used its major rival Unreal Engine for developing the VR scenes. It was also interesting to see that more recent studies did not invest in creating their own virtual environment but instead used off-the-shelf products. For those who did develop their own VR environment, the 3ds Max studio was the primary modeling tool.

### Limitations

The following limitations should be considered when interpreting the results of this review. The developed search strategy was limited to using Google Scholar for efficient and accurate search results. This may have excluded qualified articles from additional databases. In searching for studies, the terms *anxiety* and *depression* exclude studies using terms such as major depressive disorder or phobia. Moreover, because of the large number of articles reviewed, there is a possibility of overlooking valid publications that might have met the inclusion criteria. Non-English articles were not included in this review.

### Conclusions and Future Work

Most studies we reviewed demonstrated the use of VR to be effective for supporting the treatment of anxiety or depression in a range of settings and recommended its potential as a tool for use in a clinical environment. As standalone headsets are much easier to work with and more suitable for home use, the shift from tethered VR headsets to standalone headsets in the mental health environment was not observed. Nine studies explicitly mentioned the use of CBT. Out of the 9 studies, CBT was conducted within a VR environment in 5 (56%) studies, whereas in the remaining 4 (44%) studies, CBT was used as an addition to VRET. All 9 studies reported the use of CBT either in vivo or inside a virtual environment to be effective in supporting the treatment of anxiety or depression.

Although a considerable number of studies (n=34) were included in this review, some areas are still under research, thereby lowering the percentage of such studies to be included. Specifically, we found a lot of studies dedicated to one or the other form of anxiety, whereas a limited number of studies were found to concentrate on depression (4/34, 12%). Supporting people with depression in VR settings could be an interesting area to explore for health and technology researchers in the future.

Few studies conducted an RCT, as shown in Multimedia Appendix 2. Future research could use the VR scenarios and technologies outlined in this review to conduct RCTs to test the effectiveness and cost or benefits of using VR in the treatment of depression and anxiety.

Out of the 34 reviewed articles, only 9 (26%) studies explicitly mentioned the use of CBT in combination with or in addition to VRET. All 9 studies reported improvement in participants’ anxiety or depressive symptoms via CBT in addition to VRET or CBT within a virtual environment. As there is little literature on the combination of CBT and VRET, the comprehensive review produced by this study is effective in offering new insights and allows for further research on the use of CBT and VRET for a variety of mental health conditions in the future.

## Appendix

Multimedia Appendix 1 Search strategies.

Multimedia Appendix 2 A summary of reviewed articles.

Multimedia Appendix 3 Number of publications per year.

Multimedia Appendix 4 Average age group distribution.

Multimedia Appendix 5 Distribution of total participants across studies.

Multimedia Appendix 6 Number of publications per clinical conditions.

Multimedia Appendix 7 Types of virtual reality interventions.

Multimedia Appendix 8 Types of virtual reality headsets.

Multimedia Appendix 9 Number of virtual reality sessions.

## Abbreviations

CBT: cognitive behavioral therapy
HMD: head-mounted display
IE: imaginal exposure
IVE: in vivo exposure
RCT: randomized controlled trial
RQ: research question
SAD: social anxiety disorder
VR: virtual reality
VRET: virtual reality exposure therapy
WHO: World Health Organization
