# Supplementary material for: Virtual Reality for Supporting the Treatment of Depression and Anxiety: Scoping Review
Source: JMIR Ment Health. 2021 Sep 23;8(9):e29681. doi: 10.2196/29681 (PMC8498902; doi:10.2196/29681)
Supplement: Multimedia Appendix 1 [file mental_v8i9e29681_app1.docx]

**Appendix 1 – Search Strategy**

The idea was to find the most relevant papers that used virtual reality on supporting and enhancing the outcome for mental health issues, especially anxiety and depression.

We decided to use Google Scholar (<https://scholar.google.com>) as the primary source of materials included in this research. In detail, Google Scholar provides a simple way to search across a variety of disciplines, databases, and journals. To limit the potential amount of studies valid for our purposes, we used specific search criteria’s and searched terms.

The goal of our search strategy was to limit the number of results related to virtual reality in mental health to only a relevant minimum.

**Defining the keywords – search terms**

The primary search terms reflect the keywords of our research:

- Anxiety
- Depression
- Virtual Reality

Because information technology is evolving rapidly in terms of hardware and software related to virtual reality, we decided to limit the year of publishment to 2017. Therefore, the main article dates criteria were set between 2017 and 2021. Google Scholar advance search allows selecting articles using only a limited set of criteria. When we used the search filter criteria “anywhere in the article” with the text keywords “Virtual reality depression anxiety”, the Google Scholar yielded 17300 results. Thus, to process such many articles would be extremely time-consuming. Therefore, based on our search strategy goal, we decided to use a combination of three search requests, that were compliant with the following rules:

1. All keywords must be present in the article
2. All Keywords must be present in the title of the article (not only in the article body)
3. The article publish date must be within a specific range

**Figure 1.** Google Scholar advance search


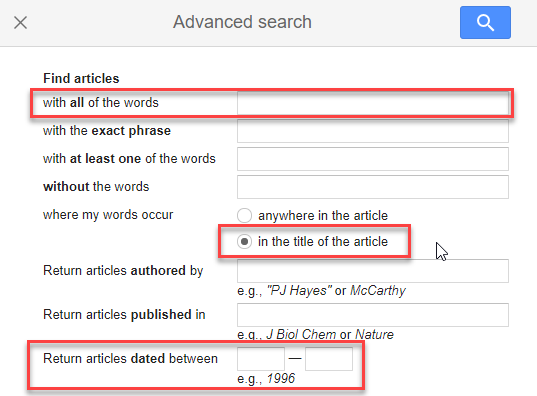


With these rules in place, we were able to execute three separate search requests using keywords:

1. “Virtual reality AND anxiety”
2. “Virtual reality AND depression”
3. “Virtual reality AND depression AND anxiety”

We got 369 article results that needed to be manually downloaded and validated before further investigation. Therefore, we proposed to develop a simple program (data collector), that would not only make the initial data validation and parsing for us but also store the data in proper structures to relational DB.

To speed up the process of gathering the data from Google Scholar, we decided to develop a python-based program, that would:

1. Create and execute three separate HTTP requests to Google Scholar based on the search criteria
2. Get the overall number of results for each request and store the HTML page of the response for each result page to local PC
3. Extract the data from each HTML page and prepare research data structures
4. Store the data structures into relational DB for further examination

**Figure 2.** Primary data search and evaluation use cases


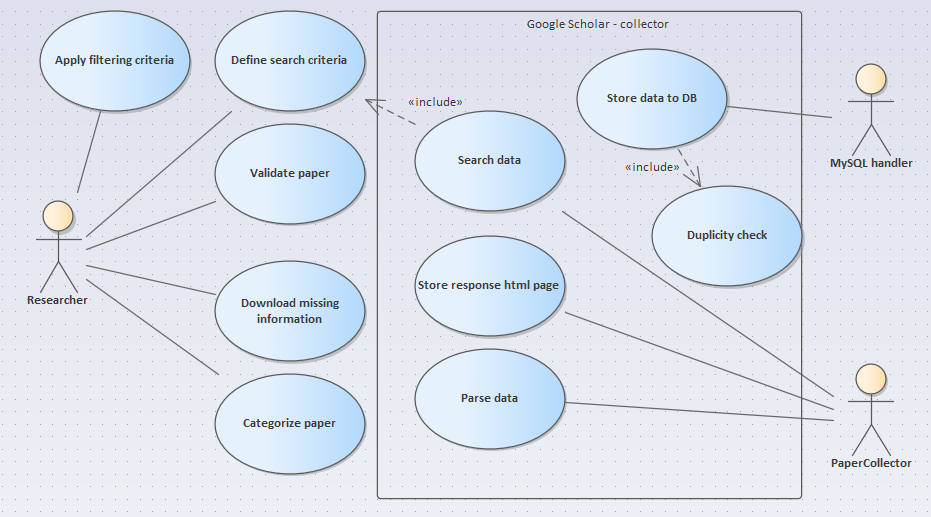


**Google Scholar**

Due to the Google Scholar policies, it is not possible to automate the search process. Still, it is possible to extract at least the data from the HTTP response of the Google Scholar page to speed-up the data evaluation process. The HTTP search request to Google Scholar API using specific parameters in the URL, especially:

*q -* query string

*as_ylo* – a year from

*as_yhi* – a year to

*as_sdt* – include patents (1 - false)

*as_vis* – include citation (1 - false)

*start* – the pagination of the result (starting page)

Therefore, it is possible to construct the URI of the request the same way as using the web page and obtain article results without violating any policies. The web scrapping process must be executed within a reasonable timeframe; otherwise, the API will block the IP address to avoid any bot activity. On the other hand, it is possible to use existing Python libraries like scholarly or other proxy implementations. Still, when we compared the effort vs value, it was not efficient for our purposes.

An example of the URL request using search criteria:

[https://scholar.google.com/scholar?start={0}&q=allintitle:+virtual+reality+anxiety&hl=en&as_sdt=1,5&as_ylo=2015&as_yhi=2020](https://scholar.google.com/scholar?start=%7b0%7d&q=allintitle:+virtual+reality+anxiety&hl=en&as_sdt=1,5&as_ylo=2015&as_yhi=2020)

**Figure 3.** Simplified data search and download activity diagram


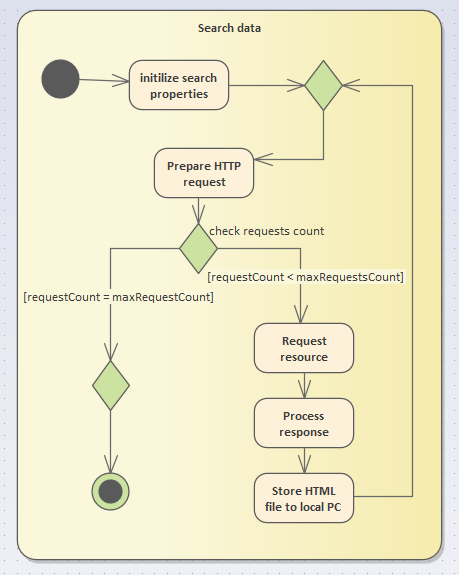


**Parsing the result**

Google Scholar returns in a single response maximum of ten articles by default with pagination information. In detail, it is possible to identify the title of the document, authors and other information by the CSS class applied on the element level in the generated HTML source code (for example *gs_rt, gs_a, gs_rs, etc.)*:

**Figure 4.** Sample of Google Scholar result page with an article


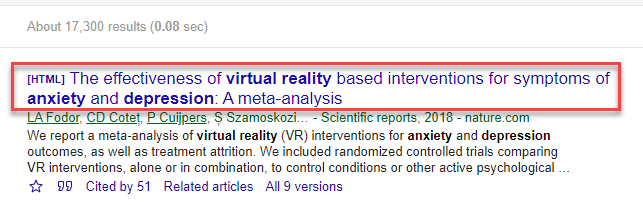


**Figure 5.** Sample of HTML source code with the article title


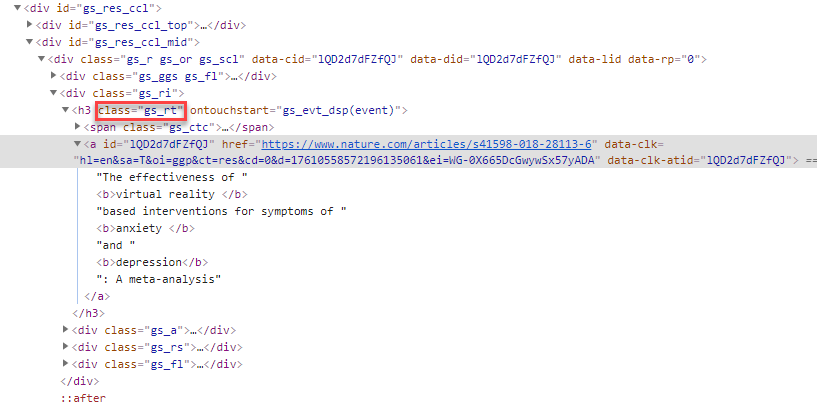


**Data parsing process**

To be able to parse the data from the HTML source code dramatically speeded up the initial data searching and evaluation process relevant for our research purposes. Nevertheless, the final stage of the data loading process was the manual validation and correction of any missing information or non-valid information.

**Figure 6.** Data collector class diagram


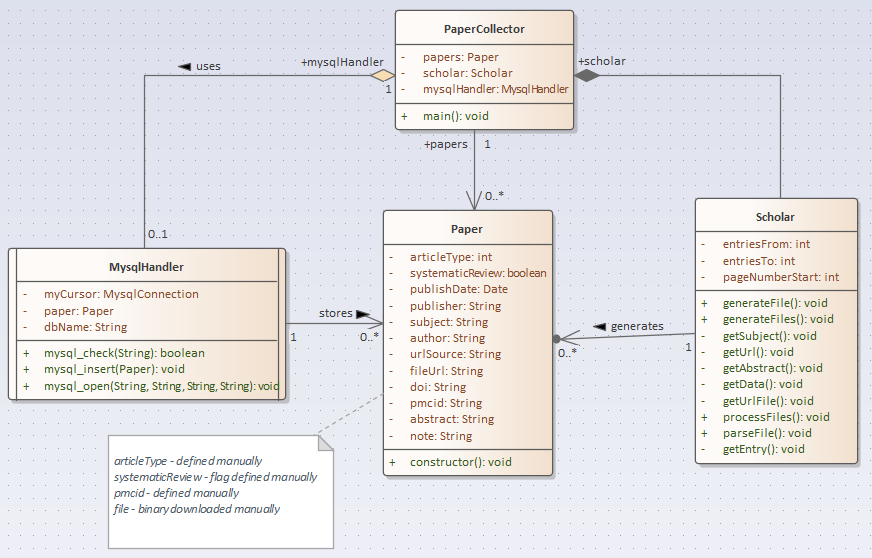


**Figure 7.** Simplified data collector parsing process diagram


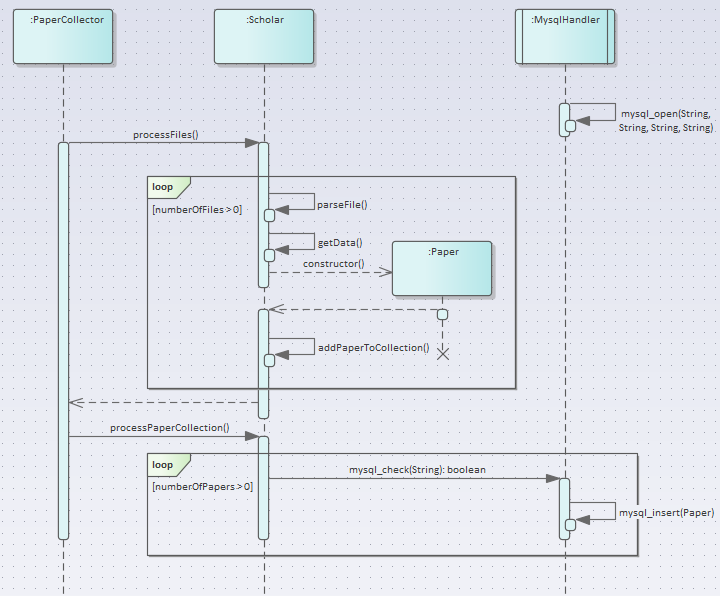


**Data sources**

We build a PHP based web site, and we used the data as the primary source of our research. It helped us in cooperation and rapidly simplified the navigation process to publisher sites and research authors.

**Figure 8.** Web site <https://andrej.kiwi/view.html>


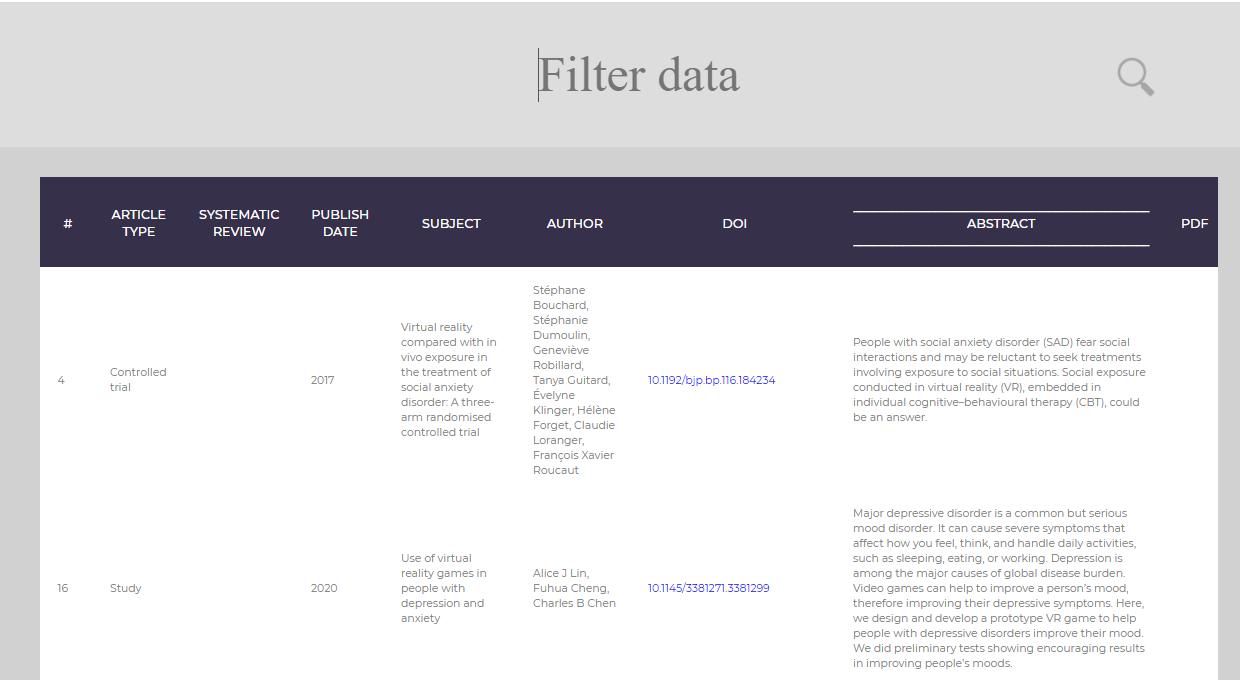


A detailed search strategy is shown in Table 1, using the search term for Google Scholar and inclusion criteria described in the above document.

**Table 1.** Electronic database search for Google Scholar

| Search ID | Search terms | Results |
| --- | --- | --- |
| **1** | \|  \|  \| \| --- \| --- \|   Virtual reality AND anxiety | 298 |
| **2** | Virtual reality AND depression | 62 |
| **3** | Virtual reality AND depression AND anxiety | 9 |
| **Total Collected** |  | 369 |
|  |  |  |
| Exclusion ID |  |  |
| **1** | excluding non-relevant studies | 53 |
| **2** | excluding duplicities and version updates | 24 |
| **3** | excluding documents with no significant reported results | 154 |
| **4** | excluding swot analysis, thesis and citations, system reviews | 27 |
| **5** | excluding documents not in the English language | 5 |
| **6** | excluding documents published before 2017 | 16 |
| **7** | excluding documents with anxiety or depression as a secondary aspect or anxiety / depression induced due to cancer, surgery, etc. | 55 |
| **8** | excluding documents with ongoing trials | 1 |
| **Total Excluded** |  | 335 |
| Final Selected |  | **34** |
