# Supplementary material for: Virtual Reality for Supporting the Treatment of Depression and Anxiety: Scoping Review
Source: JMIR Ment Health. 2021 Sep 23;8(9):e29681. doi: 10.2196/29681 (PMC8498902; doi:10.2196/29681)
Supplement: Multimedia Appendix 2 [file mental_v8i9e29681_app2.docx]

| **Author** | **Clinical Condition** | **Participants** | **Sessions** | **Intervention +**  **(Tools)** | **VR Environment** | **Key Findings** |
| --- | --- | --- | --- | --- | --- | --- |
| Anderson et al. [12] | Social Anxiety Disorder & Public Speaking Anxiety | 28 | 8 | CBT (EGT^[[1]](#endnote-1)^) & VRET | Speech in virtual conference room (n = 5), classroom (n = 35), auditorium (n = 100) | Statistically significant improvement on all self-report measures from pre-treatment to follow-up |
| Lin et al. [13] | Depression | 20 | 1 | VR Exercise + VR Games, (Head tracking and HMD^[[2]](#endnote-2)^) | Nature scenes and numerous exercises like cycling, jogging | VR games are efficient & affordable for reducing depressive symptoms and elevating mood |
| Šalkevicius et al. [23] | Anxiety and Stress | 30 | 1 | VRET^[[3]](#endnote-3)^ + Mental stress detection,  (Gear VR, wristband sensor Emaptica E4) | Virtual conference hall with audience | Classification model predicted a 4-class anxiety level with 80.1% accuracy (LOOCV^[[4]](#endnote-4)^).  It is feasible to estimate the anxiety levels of patients in VRET sessions. |
| Stamou et al. [24] | Post-Natal Depression | 6 | 6 | CBT inside VR | Participants exposed to virtual stressors, asked to tidy up the virtual middle-class 2-bedroom house | All participants reported feeling better, more relaxed and with improved mood, better self-esteem, and improved sleep and appetite at the end of therapy |
| Guitard et al. [25] | Generalized Anxiety Disorder | 28 |  | VRET & CBT,  (A 6-side CAVE^[[5]](#endnote-5)^-Like system and wireless motion tracking) | The three standardized VE^[[6]](#endnote-6)^ were  an emergency room, an apartment, a student room | The standardized VE induced significant anxiety. No difference was found between standardized VE and imagined scenario |
| Geraets et al. [26] | Generalized and Social Anxiety Disorder | 15 | 16 | CBT inside VR, (HMD Sony HMZ-T1, joystick) | A virtual street, bus, cafe and supermarket environments | Social anxiety and quality of life improved at post-treatment. At follow-up, depressive symptoms decreased, social anxiety was maintained |
| Lange & Pauli [27] | Social Anxiety Disorder | 50 | 108 | VR Task – avoidance behaviour,  (3D-multisensory 5-side CAVE) (Controlled Trial) | The virtual agent showed random idle behaviour with various facial expressions and followed the participant with gaze and body orientation | High socially anxious participants displayed enhanced avoidance behaviour towards virtual people, but no specific avoidance behaviour towards virtual people with a negative facial expression |
| Koilias et al. [28] | Car Passenger Anxiety | 45 | 1 | VR app | A tour as passengers in a virtual reality car in urban, rural VEs | Higher anxiety levels were found in a crowded urban vs less crowded rural environment |
| Zacarin et al. [29] | Public speaking anxiety | 6 | 6 | Behaviour Therapy + VRET, (Virtua Therapy Simulator, Oculus Rift) | Waiting room, classroom, auditorium | Behaviour therapy + VRET reduced public speaking anxiety and increased coping behaviours, in the virtual and non-virtual contexts |
| Kim et al. [30] | Panic Disorder & Anxiety | 7 | 2 | Visuo-haptic-based multimodal feedback VR, (Oculus CV1, Leap Motion, PSL-iECG2 heart rate monitor) | Virtual party and an elevator | Visuo-haptic VR system was effective in relieving subjective anxiety in panic disorder without comorbidity |
| Adjorlu et al. [31] | Autism Spectrum Disorder | 4 | 4 | Music Therapy + VR app, (Oculus Rift) | Sing a song in a 3D virtual concert hall | Only 1 of 3 participants reported anxiety in after study questionnaire |
| Hartanto D [32] | Social Anxiety Disorder | 5 | 10 | VRET, (eMagin Z800 HMD) | Total 19 different VEs such as meeting a blind date, a job interview, doctor’s place, talking to a stranger at a bus station and party | System evoked anxiety and patients experienced a substantial level of presence with 18 minutes of fully automated dialogues |
| Schaaf A [33] | Anxiety, Depression & Stress | 116 | 1 | Art therapy + VR app, (HTC Vive + Controllers)  (Controlled Trial) | Activity using Tilt brush VR app | No significant decrease in anxiety, depression, or stress except in affect levels across all participants |
| Suwanjatuporn & Chintakovid [34] | Depression | 5 | 3 | 360 VR Video, (Windows MRH^[[7]](#endnote-7)^ + 2 controllers) | Walking in the garden in a 360-degree view | Some positive feedback but not suitable for longer than 5 mins, Caused dizziness and vision trouble |
| Kovar I [35] | Social Anxiety Disorder | 10 | 10 | CBT inside VR,  (HTC Vive + Controllers) | Public speaking, a call from a random institution, criticism of their appearance, a job interview | Most significant improvement in the length of fluent speech. VRET improved reaction speed by 204.8s, No effect in job interview VE |
| Zinzow et al. [36] | Driving-Related Anxiety | 8 | 8 | CBT & VR Driving Simulation,  (DriveSafety CDS-250 drive simulator) | Lane Keeping Straight / Changing / Mirrors, Speed Control Straight, Pedals and Stopping, Functional Object Detection–Basic, Turning Left / Right | Hyperarousal in driving situations declined by 69%, aggressive driving declined by 29%, and risky driving declined by 21% |
| Tarrant et al. [37] | Anxiety | 12 | 1 | EEG^[[8]](#endnote-8)^ + CBT + VR Meditation,  (Gear VR) | A mindfulness meditation by Story Up^[[9]](#endnote-9)^ VR | The VR meditation significantly reduced subjectively reported anxiety and increased alpha power.  It shifted proportional power from high to low beta frequencies and reduced broadband beta activity in the anterior cingulate cortex. |
| Lewis et al. [38] | Paruresis | 9 | 1 | VRET,  (Oculus Go) | Virtual public restroom | Stress response reported to the stimulus of the virtual public toilet |
| Perandré & Haydu [39] | Social Anxiety Disorder | 2 | 17 | Behaviour analytics + VRET simulator,  (The Mindfield eSense (<https://goo.gl/kCidld>), the Vitua.Therapy VR simulator, Oculus Rift) | The scenes were divided into exposures capable of producing scrutiny anxiety, assertiveness anxiety, performance anxiety and intimacy anxiety | The simulator produced anxiety and high levels of presence during exposure; and both participants had reduced levels of anxiety at the end of intervention and generalization to the natural context |
| Nassrin Rezaie Khosravi G [40] | Anxiety | 5 | 3 | VR based Neurofeedback therapy | 3D relaxation imagery | Significant difference between the mean anxiety scores in the pre and post-test scores |
| Dehn et al. [41] | Depression | 38 | 8 | Cognitive training + Desktop VR app, (OctaVis) | Verbally memorize and buy items from VR supermarket | No greater benefit of the cognitive training as compared to desktop VR app for depression |
| Nason et al. [42] | PTSD^[[10]](#endnote-10)^ & Social Anxiety Disorder | 12 | 2 | 360 VR Video + VR Task, (Oculus Rift) | Obtain and place item in basket in large grocery store lightly populated | 360 VR video slightly more realistic, but more anxiety inducing than VR |
| Bossenbroek et al. [43] | Disruptive Classroom Behaviour & State Anxiety | 8 | NA | VR biofeedback game, (HTC Vive + DEEP belt) | Explore underwater world using breathing for movement | 6 participants showed reductions in anxiety, and 5 participants showed reductions in disruptive classroom behaviours, Calm state lasted for about 2 hours on average |
| Pallavicini & Pepe [44] | Anxiety | 36 | NA | VR games + Body involvement,  (HTC Vive) | Played AudioShield and FruitNinja VR games. | VR games elicit positive emotions decrease negative emotions and state anxiety. The level of body involvement of VR game plays an important factor in it. |
| Kim et al. [45] | Social Anxiety Disorder | 55 | 6 | VRET,  (HTC Vive, Heart rate, eye movement and skin tension monitor) | Enter a room and introduce amongst other college students while NPCs^[[11]](#endnote-11)^ reacted | Improved all elements of social anxiety measured, including general anxiety, social anxiety symptoms, and cognitive and emotional aspects of social anxiety |
| O’Meara et al. [46] | Exam Anxiety | 40 | 2 | 360 VR video + VRET,  (HTC Vive, GoPro Fusion camera)  (Controlled Trial) | A 360-degree video footage of VR Nature or VR Urban environment | VR Nature intervention had significant reductions in negative affect scores for highly anxious, four minutes exposure sufficient |
| Wang et al. [47] | Generalized Anxiety Disorder | 77 | NA | VR Exercise + EEG, (CAVE, Bicycle, ProComp Infiniti)  (RCTp) | VR cycling in VR nature or VR abstract painting environment | Cycling in VR Nature more effective than VR abstract paintings as a restorative environment to reduce stress and increase relief |
| Bouchard et al. [48] | Social Anxiety Disorder | 59 | 14 | CBT^[[12]](#endnote-12)^ inside VR^[[13]](#endnote-13)^,  (eMagin z800 HMD)  (RCT) | Workplace, Self- Introduction / Criticism scenarios | In-virtuo CBT more effective for assisting therapists than in-vivo CBT |
| Stupar-Rutenfrans et al. [49] | Public speaking anxiety | 35 | 3 | PST^[[14]](#endnote-14)^ with 360 Video + VRET,  (Smartphone) | Empty hall, small audience, large audience | PST is more effective for initial high level of speaking anxiety compared with those with moderate anxiety |
| Kim et al. [50] | Social Anxiety Disorder | 52 | 8 | Mobile VR app^[[15]](#endnote-15)^,  (Gear VR) | Conversation in 3 different social situation sets: school, business, and everyday life | Significant improvements in the total speech length, voiced-time ratio, and subjective self-ratings |
| Dechant et al. [51] | Social Anxiety Disorder | 37 | 1 | VRET + Behaviour Test  (Brain Vision Analyzer,  TobiiEyeX-Eye, 2 electrodes,  Speech recognition software) | Eight tasks in a virtual train and doctor’s waiting room scenario, the skin conductance response and gaze behaviour were monitored. | Analysing fixation durations of faces in a virtual social situation is more suitable for distinguishing low and high social-anxious participants than investigations of skin conductance responses. |
| Jeong et al. [52] | Social Anxiety Disorder | 115 | 9-11 | CBT inside VR,  (Desktop / mobile, eye movement, speaking time, heart rate monitor) | Some VEs were classroom, auditorium, job interview, train, cafe | Short-term VR-based individual CBT of 9-10 sessions may be effective. Minimal additional benefit if extended. Even 5-6 sessions can also be effective |
| Trahan et al. [53] | PTSD & Social Anxiety | 1 | 8 | Mobile VRET + EEG, (Plastic VR Headset, Brain Vision Live Amp 64-Channel) | Virtual grocery store environment | Mobile based VRET resulted in statistically significant decreases in social anxiety and PTSD and an increase sleep quality between the pre and post follow up assessment |
| Lindner et al. [54] | Public Speaking Anxiety | 23 | 1 | Routine Care VRET,  (Oculus Go) | Speech tasks in boardroom, conference, classroom VEs with customizable audience density and moods | One-session VRET in routine care effective for public speaking anxiety, delivered by CB therapists with no previous clinical experience of VR and only minimal training |

1. EGT: Exposure Group Therapy [↑](#endnote-ref-1)
2. HMD: Head Mounted Display [↑](#endnote-ref-2)
3. VRET: Virtual Reality Exposure Therapy [↑](#endnote-ref-3)
4. LOOCV: Leave-one-out Cross-Validation [↑](#endnote-ref-4)
5. CAVE: Cave Automatic Virtual Environment [↑](#endnote-ref-5)
6. VE: Virtual Environment [↑](#endnote-ref-6)
7. MRH: Mixed Reality Headset [↑](#endnote-ref-7)
8. EEG: Electroencephalogram [↑](#endnote-ref-8)
9. Story Up VR, Columbia, MO, United States [↑](#endnote-ref-9)
10. PTSD: Post-traumatic stress disorder [↑](#endnote-ref-10)
11. NPCs: Non-Playable Characters [↑](#endnote-ref-11)
12. CBT: Cognitive Behaviour Therapy [↑](#endnote-ref-12)
13. VR: Virtual Reality [↑](#endnote-ref-13)
14. PST: Public Speech Trainer [↑](#endnote-ref-14)
15. App: Application

    p RCT: Randomised Controlled Trial [↑](#endnote-ref-15)
