# Supplementary figures and images for: Virtual Reality for Supporting the Treatment of Depression and Anxiety: Scoping Review
Source: JMIR Ment Health. 2021 Sep 23;8(9):e29681. doi: 10.2196/29681 (PMC8498902; doi:10.2196/29681)

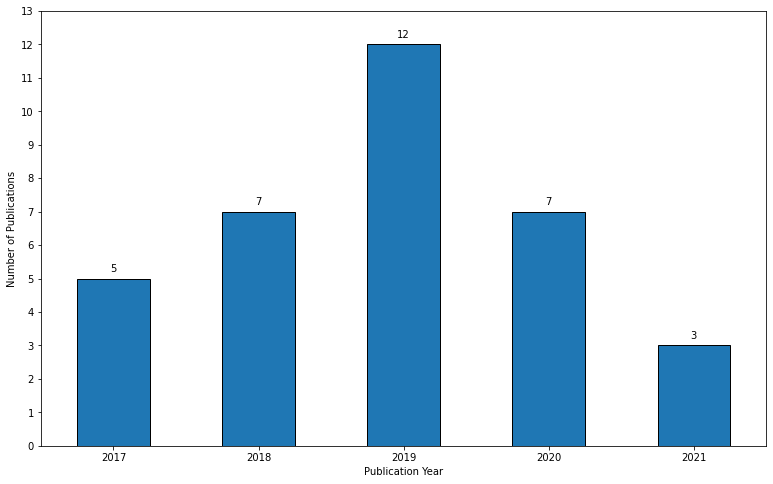

Supplement: Multimedia Appendix 3 [file mental_v8i9e29681_app3.png]

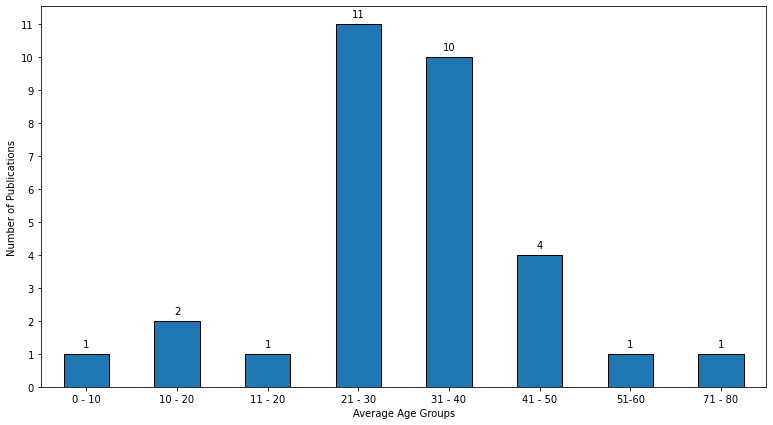

Supplement: Multimedia Appendix 4 [file mental_v8i9e29681_app4.png]

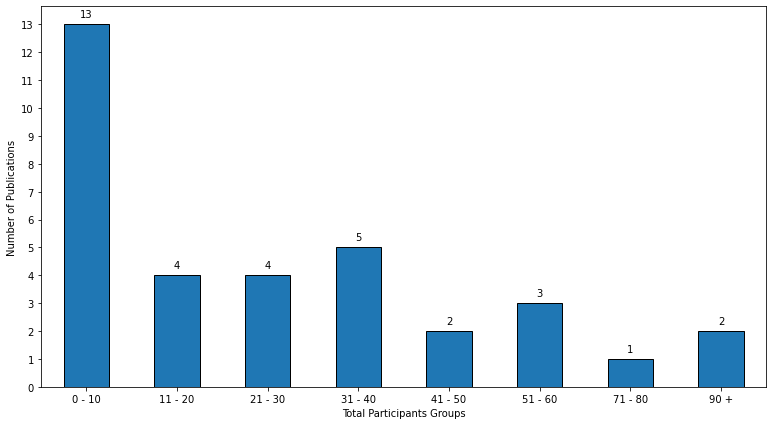

Supplement: Multimedia Appendix 5 [file mental_v8i9e29681_app5.png]

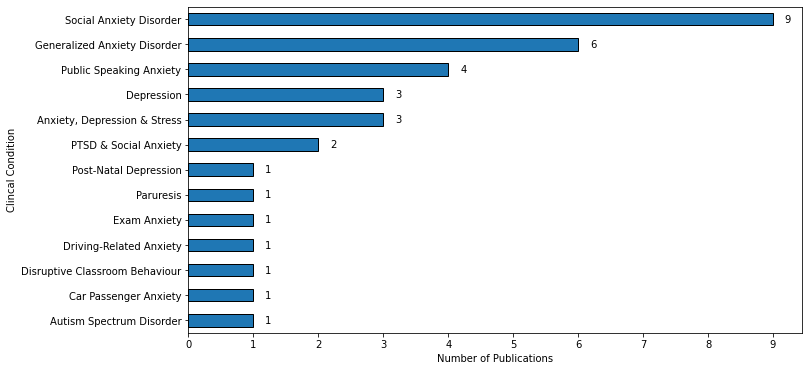

Supplement: Multimedia Appendix 6 [file mental_v8i9e29681_app6.png]

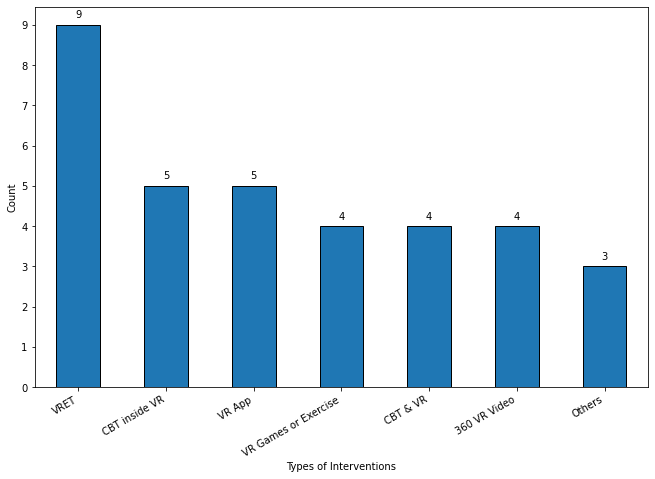

Supplement: Multimedia Appendix 7 [file mental_v8i9e29681_app7.png]

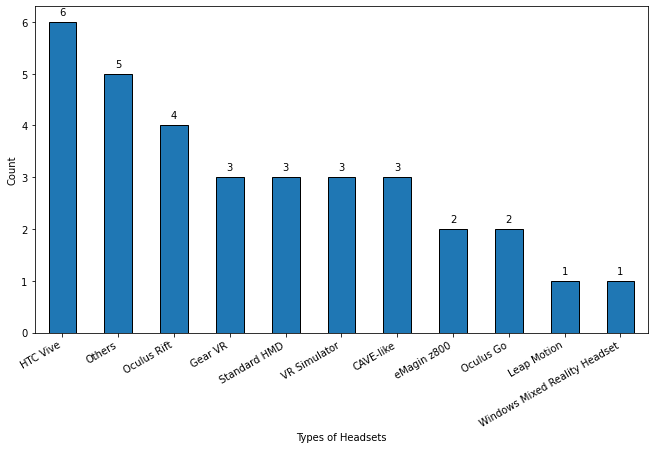

Supplement: Multimedia Appendix 8 [file mental_v8i9e29681_app8.png]

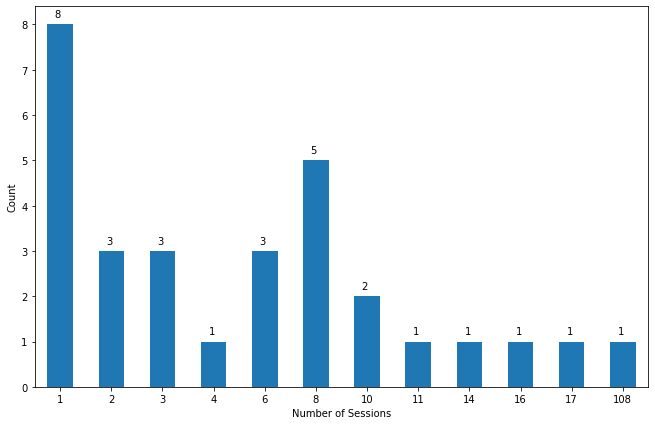

Supplement: Multimedia Appendix 9 [file mental_v8i9e29681_app9.png]
